# 2-[(*E*)-(5-Chloro-2-methyl­phen­yl)imino­meth­yl]-4-methyl­phenol

**DOI:** 10.1107/S1600536813019454

**Published:** 2013-07-27

**Authors:** Yun-Fa Zheng

**Affiliations:** a Department of Chemistry, Lishui University, Lishui 323000, People’s Republic of China

## Abstract

In the mol­ecule of the title Schiff base compound, C_15_H_14_ClNO, the two benzene rings are twisted with respect to each other, with a dihedral angle of 35.0 (3)°; an intra­molecular O—H⋯N hydrogen bond occurs. In the crystal, weak C—H⋯π inter­actions between methyl groups and chloro­phenyl rings link the mol­ecules into supra­molecular chains running along the *a* axis.

## Related literature

For background to phyenyl­amine compounds, see: Fan *et al.* (2012 ▶). For applications of Schiff base derivatives, see: Siddiqui *et al.* (2006 ▶); Ebrahimipour *et al.* (2012 ▶).
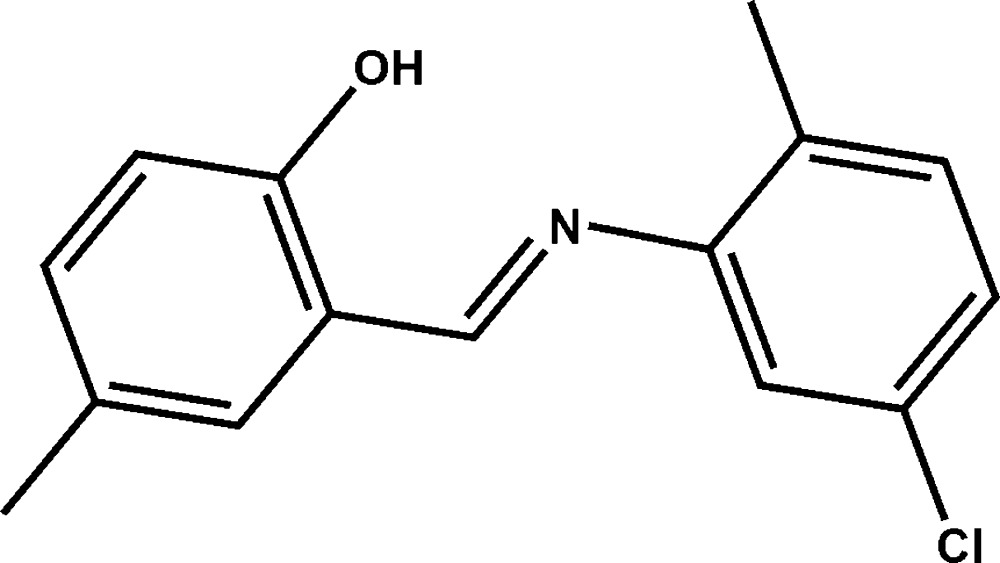


## Experimental

### 

#### Crystal data

C_15_H_14_ClNO
*M* *_r_* = 259.72
Orthorhombic, 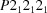

*a* = 7.6629 (10) Å
*b* = 11.8442 (14) Å
*c* = 14.342 (2) Å
*V* = 1301.7 (3) Å^3^
*Z* = 4
Mo *K*α radiation
μ = 0.28 mm^−1^
*T* = 293 K
0.45 × 0.42 × 0.35 mm

#### Data collection

Agilent Xcalibur Gemini ultra diffractometer with Atlas detector
Absorption correction: multi-scan (*CrysAlis PRO*; Agilent, 2010 ▶) *T*_min_ = 0.88, *T*_max_ = 0.91
8663 measured reflections
2382 independent reflections
1769 reflections with *I* > 2σ(*I*)
*R*_int_ = 0.048

#### Refinement

*R*[*F* ^2^ > 2σ(*F* ^2^)] = 0.041
*wR*(*F* ^2^) = 0.100
*S* = 1.03
2382 reflections
169 parameters
H atoms treated by a mixture of independent and constrained refinement
Δρ_max_ = 0.16 e Å^−3^
Δρ_min_ = −0.17 e Å^−3^
Absolute structure: Flack (1983 ▶), with 991 Friedel pairs
Absolute structure parameter: −0.18 (9)

### 

Data collection: *CrysAlis PRO* (Agilent, 2010 ▶); cell refinement: *CrysAlis PRO*; data reduction: *CrysAlis PRO*; program(s) used to solve structure: *SHELXTL* (Sheldrick, 2008 ▶); program(s) used to refine structure: *SHELXTL*; molecular graphics: *SHELXTL*; software used to prepare material for publication: *SHELXTL*.

## Supplementary Material

Crystal structure: contains datablock(s) I, global. DOI: 10.1107/S1600536813019454/xu5720sup1.cif

Structure factors: contains datablock(s) I. DOI: 10.1107/S1600536813019454/xu5720Isup2.hkl

Supplementary material file. DOI: 10.1107/S1600536813019454/xu5720Isup3.cml

Additional supplementary materials:  crystallographic information; 3D view; checkCIF report

## Figures and Tables

**Table 1:** Hydrogen-bond geometry (Å, °) *Cg*1 is the centroid of the C9–C14 benzene ring.

*D*—H⋯*A*	*D*—H	H⋯*A*	*D*⋯*A*	*D*—H⋯*A*
O1—H1⋯N1	0.84 (4)	1.84 (4)	2.629 (3)	154 (4)
C7—H7*C*⋯*Cg*1^i^	0.96	2.91	3.767 (3)	149

Symmetry code: (i) 
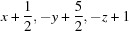
.

**Fig. 1.**
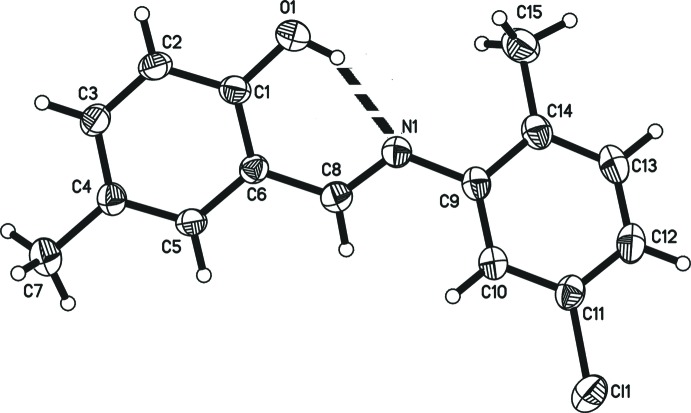
Thermal ellipsoid plot of the title compound. Ellipsoids are drawn at the 50% probability level and H atoms are represented as small spheres of arbitrary radius. The dashed line indicates the intramolecular hydrogen bond.

**Fig. 2.**
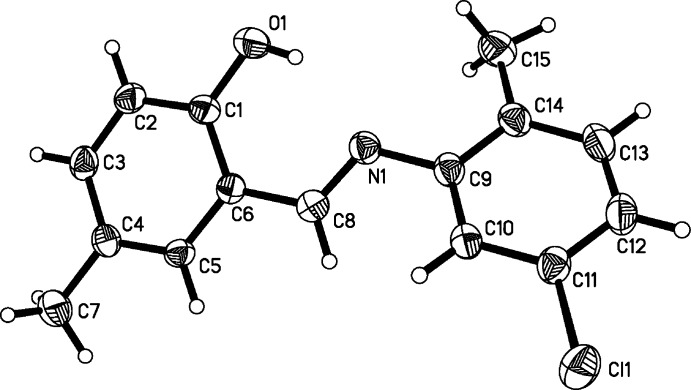
Thermal ellipsoid plot of the title compound. Ellipsoids are drawn at the 50% probability level and H atoms are represented as small spheres of arbitrary radius.

## supplementary crystallographic information

### Comment

Phyenylamine group has received attention in recent years for their unique physical and chemical properties (Fan *et al.*, 2012). On the other hand, Schiff bases derived from salicyladehyde and methylaniline with varous substituents have exhibited potential application in pharmaceutical field for their properties of antitumor, antimicrobial and antiviral activities (Siddiqui *et al.*, 2006). Moreover, Schiff bases ligands are potentially capable of forming stable complexes by coordination of metal ions with their nitrogen donors (Ebrahimipour *et al.*, 2012). As an extension work on the structural characterization of Schiff base compounds, the title compound is reported.

The molecular structure of title compound shows an E configuration, with a C9—N1═C8—C6 torsion angle of 0.26 (4)°. The bond distance of N1═C8 at 1.284 (3)Å is a typical double bond. It is noteworthy that H1 atom bonded to O1 is involved in an O1—H1···N1 intramolecular hydrogen bond, which results in formation of a six-membered ring (Fig. 1). The dihedral angle between the two planes of the chlorophyenyl ring and methylphenol ring is 35.0 (3)°.

### Experimental

A mixture of 5-chloro-2-methylaniline (1.42g, 10.0 mmol), 3-methyl-2-hydroxybenzaldehyde (1.36g, 10.0 mmol) in 50.0 ml CH_2_Cl_2_ was fluxed under an Ar atmosphere for about 6 h to gain a yellow precipitate. The product was collected by filtration and washed with cold ethanol to give the Schiff base compound (2.25g in 86% yield). The yellow single crystals suitable for X-ray analysis were crystallized from CH_2_Cl_2_/absolute ethanol (3/2) systems by slow evaporation of solvents at room temperature over a week. Elemental analysis. Calc. for C_15_H_14_ClNO: C 69.36, H, 6.43%; Found C 69.92, H 6.80%.

### Refinement

Hydroxy H atom was located in a difference Fourier map and positional parameters were refined freely, U_iso_(H) = 1.5U_eq_(O). Other H atoms were fixed geometrically and treated as riding with C—H = 0.96 Å (methyl) or 0.93 Å (aromatic), U_iso_(H) = 1.2U_eq_(C) for aromatic H atoms or U_iso_(H) = 1.5U_eq_(C) for methyl H atoms.

### Crystal data

**Table:**

C_15_H_14_ClNO	*F*(000) = 544
*M**_r_* = 259.72	*D*_x_ = 1.325 Mg m^−^^3^
Orthorhombic, *P*2_1_2_1_2_1_	Mo *K*α radiation, λ = 0.71073 Å
Hall symbol: P 2ac 2ab	Cell parameters from 1894 reflections
*a* = 7.6629 (10) Å	θ = 3.4–29.6°
*b* = 11.8442 (14) Å	µ = 0.28 mm^−^^1^
*c* = 14.342 (2) Å	*T* = 293 K
*V* = 1301.7 (3) Å^3^	Block, yellow
*Z* = 4	0.45 × 0.42 × 0.35 mm

### Data collection

**Table:**

Agilent Xcalibur Gemini ultra diffractometer with Atlas detector	2382 independent reflections
Radiation source: fine-focus sealed tube	1769 reflections with *I* > 2σ(*I*)
Graphite monochromator	*R*_int_ = 0.048
Detector resolution: 10.3592 pixels mm^-1^	θ_max_ = 25.4°, θ_min_ = 3.4°
ω scans	*h* = −9→7
Absorption correction: multi-scan (*CrysAlis PRO*; Agilent, 2010)	*k* = −14→14
*T*_min_ = 0.88, *T*_max_ = 0.91	*l* = −15→17
8663 measured reflections	

### Refinement

**Table:**

Refinement on *F*^2^	Hydrogen site location: inferred from neighbouring sites
Least-squares matrix: full	H atoms treated by a mixture of independent and constrained refinement
*R*[*F*^2^ > 2σ(*F*^2^)] = 0.041	*w* = 1/[σ^2^(*F*_o_^2^) + (0.0463*P*)^2^] where *P* = (*F*_o_^2^ + 2*F*_c_^2^)/3
*wR*(*F*^2^) = 0.100	(Δ/σ)_max_ = 0.002
*S* = 1.03	Δρ_max_ = 0.16 e Å^−^^3^
2382 reflections	Δρ_min_ = −0.17 e Å^−^^3^
169 parameters	Extinction correction: *SHELXTL* (Sheldrick, 2008), Fc^*^=kFc[1+0.001xFc^2^λ^3^/sin(2θ)]^-1/4^
0 restraints	Extinction coefficient: 0.024 (2)
Primary atom site location: structure-invariant direct methods	Absolute structure: Flack (1983), with 991 Friedel pairs
Secondary atom site location: difference Fourier map	Flack parameter: −0.18 (9)

### Special details

**Table:**

Geometry. All esds (except the esd in the dihedral angle between two l.s. planes) are estimated using the full covariance matrix. The cell esds are taken into account individually in the estimation of esds in distances, angles and torsion angles; correlations between esds in cell parameters are only used when they are defined by crystal symmetry. An approximate (isotropic) treatment of cell esds is used for estimating esds involving l.s. planes.
Refinement. Refinement of F^2^ against ALL reflections. The weighted R-factor wR and goodness of fit S are based on F^2^, conventional R-factors R are based on F, with F set to zero for negative F^2^. The threshold expression of F^2^ > 2sigma(F^2^) is used only for calculating R-factors(gt) etc. and is not relevant to the choice of reflections for refinement. R-factors based on F^2^ are statistically about twice as large as those based on F, and R-factors based on ALL data will be even larger.

### Fractional atomic coordinates and isotropic or equivalent isotropic displacement parameters (Å^2^)

**Table:**

	*x*	*y*	*z*	*U*_iso_*/*U*_eq_	
Cl1	0.87007 (10)	0.98698 (7)	0.87968 (5)	0.0705 (3)	
O1	1.1714 (3)	1.01909 (17)	0.36679 (14)	0.0651 (6)	
N1	1.0171 (3)	0.99976 (18)	0.52972 (14)	0.0456 (5)	
C1	1.1228 (3)	1.1295 (2)	0.36948 (19)	0.0449 (6)	
C2	1.1680 (4)	1.1989 (2)	0.2958 (2)	0.0510 (7)	
H2	1.2327	1.1705	0.2462	0.061*	
C3	1.1167 (4)	1.3109 (2)	0.29627 (19)	0.0498 (7)	
H3	1.1449	1.3561	0.2454	0.060*	
C4	1.0248 (3)	1.3579 (2)	0.36974 (19)	0.0451 (7)	
C5	0.9829 (3)	1.2883 (2)	0.44320 (19)	0.0421 (6)	
H5	0.9215	1.3181	0.4934	0.050*	
C6	1.0296 (3)	1.1738 (2)	0.44475 (18)	0.0395 (6)	
C7	0.9729 (4)	1.4803 (2)	0.3692 (2)	0.0653 (8)	
H7A	0.8860	1.4932	0.4162	0.098*	
H7B	0.9261	1.4996	0.3092	0.098*	
H7C	1.0733	1.5263	0.3818	0.098*	
C8	0.9758 (3)	1.1045 (2)	0.52278 (19)	0.0458 (7)	
H8	0.9087	1.1370	0.5697	0.055*	
C9	0.9585 (3)	0.9368 (2)	0.6085 (2)	0.0446 (7)	
C10	0.9508 (3)	0.9850 (2)	0.69650 (18)	0.0459 (6)	
H10	0.9883	1.0589	0.7055	0.055*	
C11	0.8874 (4)	0.9232 (2)	0.77061 (19)	0.0493 (7)	
C12	0.8366 (4)	0.8127 (3)	0.7589 (2)	0.0596 (8)	
H12	0.7943	0.7710	0.8089	0.071*	
C13	0.8498 (4)	0.7652 (2)	0.6719 (2)	0.0593 (8)	
H13	0.8168	0.6901	0.6644	0.071*	
C14	0.9102 (3)	0.8243 (2)	0.5945 (2)	0.0503 (7)	
C15	0.9174 (4)	0.7697 (2)	0.5002 (2)	0.0680 (9)	
H15A	1.0351	0.7715	0.4773	0.102*	
H15B	0.8427	0.8099	0.4579	0.102*	
H15C	0.8791	0.6927	0.5050	0.102*	
H1	1.136 (5)	0.993 (3)	0.418 (3)	0.102*	

### Atomic displacement parameters (Å^2^)

**Table:**

	*U*^11^	*U*^22^	*U*^33^	*U*^12^	*U*^13^	*U*^23^
Cl1	0.0837 (5)	0.0789 (6)	0.0487 (5)	0.0012 (4)	0.0059 (4)	0.0083 (4)
O1	0.0850 (14)	0.0494 (12)	0.0608 (14)	0.0107 (11)	0.0181 (11)	−0.0039 (11)
N1	0.0509 (12)	0.0429 (12)	0.0430 (13)	−0.0007 (12)	−0.0008 (10)	0.0014 (11)
C1	0.0489 (14)	0.0421 (14)	0.0439 (17)	−0.0003 (12)	0.0010 (14)	−0.0065 (13)
C2	0.0581 (17)	0.0534 (17)	0.0414 (16)	−0.0070 (14)	0.0103 (14)	−0.0092 (14)
C3	0.0590 (16)	0.0519 (16)	0.0386 (16)	−0.0127 (16)	0.0026 (14)	0.0014 (13)
C4	0.0463 (14)	0.0429 (14)	0.0460 (18)	−0.0033 (12)	−0.0052 (13)	0.0036 (13)
C5	0.0442 (14)	0.0442 (14)	0.0378 (16)	−0.0006 (12)	0.0034 (12)	−0.0058 (12)
C6	0.0409 (13)	0.0416 (14)	0.0360 (16)	−0.0039 (12)	−0.0008 (12)	−0.0009 (12)
C7	0.0764 (19)	0.0512 (16)	0.068 (2)	0.0039 (16)	0.0027 (17)	0.0091 (17)
C8	0.0445 (15)	0.0480 (15)	0.0447 (18)	−0.0003 (13)	0.0003 (13)	−0.0012 (13)
C9	0.0411 (14)	0.0421 (14)	0.0505 (19)	0.0006 (11)	−0.0003 (13)	0.0066 (13)
C10	0.0464 (14)	0.0417 (13)	0.0497 (17)	−0.0017 (13)	−0.0003 (12)	0.0056 (14)
C11	0.0475 (16)	0.0528 (16)	0.0475 (18)	0.0044 (14)	0.0002 (14)	0.0111 (13)
C12	0.0596 (19)	0.0546 (19)	0.064 (2)	−0.0002 (15)	0.0067 (15)	0.0199 (16)
C13	0.0618 (19)	0.0399 (15)	0.076 (2)	−0.0021 (14)	0.0013 (17)	0.0095 (16)
C14	0.0469 (16)	0.0437 (15)	0.060 (2)	0.0048 (13)	−0.0010 (14)	0.0052 (14)
C15	0.075 (2)	0.0530 (17)	0.076 (2)	−0.0040 (16)	0.0029 (17)	−0.0109 (17)

### Geometric parameters (Å, º)

**Table:**

Cl1—C11	1.742 (3)	C7—H7B	0.9600
O1—C1	1.361 (3)	C7—H7C	0.9600
O1—H1	0.84 (4)	C8—H8	0.9300
N1—C8	1.284 (3)	C9—C10	1.387 (4)
N1—C9	1.426 (3)	C9—C14	1.397 (3)
C1—C2	1.383 (4)	C10—C11	1.379 (3)
C1—C6	1.396 (3)	C10—H10	0.9300
C2—C3	1.383 (4)	C11—C12	1.376 (4)
C2—H2	0.9300	C12—C13	1.372 (4)
C3—C4	1.384 (4)	C12—H12	0.9300
C3—H3	0.9300	C13—C14	1.392 (4)
C4—C5	1.376 (3)	C13—H13	0.9300
C4—C7	1.503 (4)	C14—C15	1.501 (4)
C5—C6	1.403 (3)	C15—H15A	0.9600
C5—H5	0.9300	C15—H15B	0.9600
C6—C8	1.448 (3)	C15—H15C	0.9600
C7—H7A	0.9600		
			
C1—O1—H1	104 (3)	N1—C8—H8	118.8
C8—N1—C9	119.3 (2)	C6—C8—H8	118.8
O1—C1—C2	118.8 (2)	C10—C9—C14	120.8 (2)
O1—C1—C6	121.5 (2)	C10—C9—N1	121.2 (2)
C2—C1—C6	119.7 (2)	C14—C9—N1	117.9 (2)
C1—C2—C3	119.7 (3)	C11—C10—C9	119.8 (2)
C1—C2—H2	120.2	C11—C10—H10	120.1
C3—C2—H2	120.2	C9—C10—H10	120.1
C2—C3—C4	122.3 (2)	C12—C11—C10	120.7 (3)
C2—C3—H3	118.9	C12—C11—Cl1	120.1 (2)
C4—C3—H3	118.9	C10—C11—Cl1	119.3 (2)
C5—C4—C3	117.4 (2)	C13—C12—C11	118.7 (3)
C5—C4—C7	121.3 (2)	C13—C12—H12	120.6
C3—C4—C7	121.3 (2)	C11—C12—H12	120.6
C4—C5—C6	122.1 (2)	C12—C13—C14	122.9 (3)
C4—C5—H5	118.9	C12—C13—H13	118.6
C6—C5—H5	118.9	C14—C13—H13	118.6
C1—C6—C5	118.7 (2)	C13—C14—C9	117.0 (3)
C1—C6—C8	122.1 (2)	C13—C14—C15	121.0 (2)
C5—C6—C8	119.2 (2)	C9—C14—C15	122.0 (3)
C4—C7—H7A	109.5	C14—C15—H15A	109.5
C4—C7—H7B	109.5	C14—C15—H15B	109.5
H7A—C7—H7B	109.5	H15A—C15—H15B	109.5
C4—C7—H7C	109.5	C14—C15—H15C	109.5
H7A—C7—H7C	109.5	H15A—C15—H15C	109.5
H7B—C7—H7C	109.5	H15B—C15—H15C	109.5
N1—C8—C6	122.5 (2)		

### Hydrogen-bond geometry (Å, º)

Cg1 is the centroid of the C9–C14 benzene ring.

**Table:**

*D*—H···*A*	*D*—H	H···*A*	*D*···*A*	*D*—H···*A*
O1—H1···N1	0.84 (4)	1.84 (4)	2.629 (3)	154 (4)
C7—H7*C*···*Cg*1^i^	0.96	2.91	3.767 (3)	149

Symmetry code: (i) *x*+1/2, −*y*+5/2, −*z*+1.
